# Involvement of a Rarely Used Splicing SD2b Site in the Regulation of HIV-1 *vif* mRNA Production as Revealed by a Growth-Adaptive Mutation

**DOI:** 10.3390/v15122424

**Published:** 2023-12-14

**Authors:** Takaaki Koma, Naoya Doi, Bao Quoc Le, Tomoyuki Kondo, Mitsuki Ishizue, Chiaki Tokaji, Chizuko Tsukada, Akio Adachi, Masako Nomaguchi

**Affiliations:** 1 Department of Microbiology, Graduate School of Medicine, Tokushima University, Tokushima 770-8503, Japan; tkoma@tokushima-u.ac.jp (T.K.); naoya@tokushima-u.ac.jp (N.D.); baole@ump.edu.vn (B.Q.L.); kondo@tokushima-u.ac.jp (T.K.);; 2 Faculty of Medicine, Tokushima University, Tokushima 770-8503, Japan

**Keywords:** HIV-1, Vif, splicing, adaptation, APOBEC3, SD2b, SA1

## Abstract

We have previously reported an HIV-1 mutant designated NL-Y226tac that expresses Vif at an ultra-low level, being replication-defective in high-APOBEC3G cells, such as H9. It carries a synonymous mutation within the splicing SA1 site relative to its parental clone. In order to determine whether a certain mutant(s) emerges during multi-infection cycles, we maintained H9 cells infected with a relatively low or high input of NL-Y226tac for extended time periods. Unexpectedly, we reproducibly identified a g5061a mutation in the SD2b site in the two independent long-term culture experiments that partially increases Vif expression and replication ability. Importantly, the adaptive mutation g5061a was demonstrated to enhance *vif* mRNA production by activation of the SA1 site mediated through increasing usage of a rarely used SD2b site. In the long-term culture initiated by a high virus input, we additionally found a Y226Fttc mutation at the original Y226tac site in SA1 that fully restores Vif expression and replication ability. As expected, the adaptive mutation Y226Fttc enhances *vif* mRNA production through increasing the splicing site usage of SA1. Our results here revealed the importance of the SD2b nucleotide sequence in producing *vif* mRNA involved in the HIV-1 adaptation and of mutual antagonism between Vif and APOBEC3 proteins in HIV-1 adaptation/evolution and survival.

## 1. Introduction

Viruses must survive in given environments while mutating and adapting under certain selective pressures, such as the host’s restriction factors and immune system. The high mutation and adaptation abilities of viruses, especially RNA viruses, enables the emergence of unprecedented viruses including vaccine/immune-escape mutants, drug-resistant mutants, and mutants that alter replication ability, pathogenicity, and even host range [1,2,3,4,5]. Many mutant viruses, such as drug-resistant HIV-1 strains and SARS-CoV-2 circulating variant strains, have nonsynonymous mutations that result in amino acid substitutions [1,2,3,4,5]. Such nonsynonymous mutations can change the structure, activity, and function of viral proteins and allow viruses to adapt to environments. On the other hand, synonymous mutations without amino acid substitutions in viral genomes can also affect various biological processes (transcription, RNA structure, splicing, translation, microRNA targeting, etc.) [6,7,8]. Viruses including influenza virus and poliovirus have been shown to be attenuated by introducing plenty of synonymous mutations into their genomes [6,7,8]. It implies that synonymous mutations are not always random or neutral for virus phenotypes, having virological significance.

Synonymous (silent) mutations in the HIV-1 genome can affect replication ability and viral phenotypes [8]. It has been shown that HIV-1 viruses generated by global silent mutagenesis were divided into three groups with distinct phenotypes: (1) replication-competent-like wild type (WT); (2) replication defects with aberrant splicing; (3) replication defects without splicing perturbation [9]. HIV-1 produces various mRNAs encoding viral proteins through alternative splicing, which utilizes at least four splicing donor sites (SD1–SD4) and at least seven splicing acceptor sites (SA1–SA7) in its genome [10,11,12,13]. More than 50 mRNA species have been shown to be produced during HIV-1 gene expression. HIV-1 mRNAs production is regulated not only by the splicing sites (SD1–SD4 and SA1–SA7) but also by the other splicing sites like the rarely used SD2b, various splicing regulatory elements (SREs), and host proteins that recognize and bind to these *cis*-acting elements [14,15,16]. Thus, changes in the nucleotide sequence of these *cis*-acting elements can vary replication potential by affecting HIV-1 gene expression.

**Figure 1:**
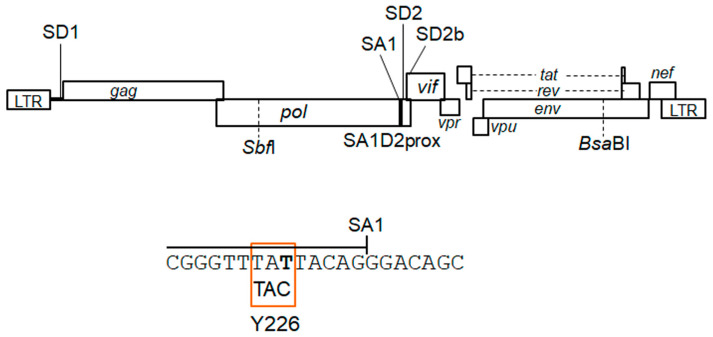
HIV-1 genome organization. Of the splicing sites on the HIV-1 genome, only SD1, SA1, SD2, and SD2b sites important for the *vif* mRNA production are presented. The black square shows SA1D2prox that we previously identified as a regulatory region involved in the alteration of the *vif* mRNA/Vif protein expression levels. Restriction enzyme sites (*Sbf*I and *Bsa*BI) used to generate adaptive proviral clones are indicated. Nucleotide sequence around the SA1 and Y226tac mutation sites are highlighted.

HIV-1 Vif antagonizes an intrinsic host restriction factor, APOBEC3 proteins, through proteasomal degradation and thus is essential for viral replication in target cells including CD4-positive T cells and macrophages [17,18,19,20,21,22]. In the absence of Vif, APOBEC3 proteins, especially APOBEC3G protein (A3G), introduce lethal mutations in the HIV-1 genome by their cytidine deaminase activity [17,18,19,20,21,22]. APOBEC3 proteins have been shown to exhibit other activities and functions that negatively affect HIV-1 replication in a deaminase-independent manner [23]. The disruption of power balance between Vif and APOBEC3 proteins that resulted from their quantitative and/or qualitative alteration can vary HIV-1 growth ability in a virologically significant manner. HIV-1 *vif* mRNA is produced by splicing at SD1 and SA1 (Figure 1) [10,11,12,13]. Activation of splicing at the SA1 site is proceeded by the binding of U1snRNP to the downstream SD2 or SD2b site, and then subsequent recognition of the SA1 site by U2snRNP completes the exon definition [24]. Although SREs such as ESEVif and ESS2b involved in *vif* mRNA production have been identified [24,25,26,27], the overall regulatory mechanism for *vif* mRNA production is very complicated and remains to be elucidated. Based on our HIV-1 adaptation experiments and sequence analysis using the HIV-1 sequence database (https://www.hiv.lanl.gov/content/index, accessed on 7 June 2023), we have successfully demonstrated that several naturally occurring synonymous single-nucleotide mutations (nsSNMs) within the region around the SA1–SD2 sites actually fluctuate *vif* mRNA/Vif protein expression levels (the SA1D2prox region in Figure 1) [28,29,30]. This finding implies that nsSNMs within the SA1D2prox region that modulate Vif expression levels are associated with the HIV-1 adaptation.

We have previously reported an ultra-low Vif-type HIV-1 clone (NL-Y226tac) that carries an nsSNM in the SA1 site located in the polymerase-integrase (Pol-IN) region in its genome (Figure 1) [28,29,30]. Virus growth of the NL-Y226tac clone was severely attenuated in a CD4-positive lymphocyte cell line H9 expressing a high level of A3G [28,29,30]. In this study, through the HIV-1 adaptation experiments in H9 cells, we investigated the following: (1) whether an ultra-low Vif-type HIV-1 clone can adapt itself to an environment under replication-restrictive conditions imposed by cells expressing high levels of A3G; (2) whether the clone acquires an adapted mutation(s) that increase Vif expression levels during the adaptation process; (3) if (2) stands, then how the adaptive mutation contributes to enhancing the Vif expression. We aimed to gain insights into splicing regulation sites that are implicated in *vif* mRNA production as well as HIV-1 adaptation.

## 2. Materials and Methods

### 2.1. Plasmid DNA

Proviral clones pNL4-3 [31], pNL-IN-Y226tac (Y226tac) [28,29,30], and pcNLmini-RI [32] have been described previously. The introduction of mutations into these clones was done by PCR-based site-directed mutagenesis. Cloning of the region from *vpu* to *env* of an adapted virus clone into pNL4-3 was carried out using unique restriction enzyme sites (*Eco*RI in the *vpr* gene and *Xho*I in the *nef* gene) in their genomes.

### 2.2. Cells

HEK293T [33] and TZM-bl [34,35] cell lines were cultured in Eagle’s MEM containing 10% heat-inactivated fetal bovine serum. Human lymphocytic H9 cells were maintained in RPMI1640 containing 10% heat-inactivated fetal bovine serum.

### 2.3. Adaptation Experiments

**Figure 2:**
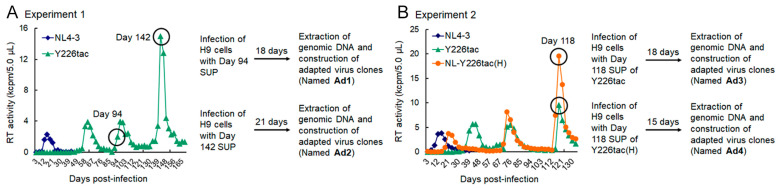
Long-term cultures of H9 cells infected with NL4-3 or its ultra-low Vif-type mutant Y226tac to obtain adapted virus clones. (**A**,**B**) Virus samples were prepared from HEK293T cells transfected with the indicated clones and inoculated into H9 cells (10^4^, 10^5^, and 10^6^ RT units for NL4-3, Y226tac, and Y226tac(H), respectively, into 10^6^ cells). Culture media of infected cells were replaced every 3 days and fresh H9 cells were added to the cultures to promote viral growth when RT activity in the supernatants was decreased to the background level. To generate adapted virus clones, H9 cells inoculated with the culture supernatants indicated were collected and subjected to proviral DNA preparations as described.

Proviral clones were transfected into HEK293T cells by the calcium phosphate coprecipitation method [31,36] to prepare virus samples for infection experiments. The virion-associated reverse transcriptase (RT) activity was measured as previously described [37,38] to quantify virus amounts. Virus samples were inoculated into H9 cells and the infected cells were maintained as the long-term culture in Figure 2 as previously described [32]. Potentially adapted proviral clones derived from the long-term culture were constructed by introducing the PCR-amplified fragments from *Sbf*I to *Bsa*BI sites (Figure 1) into the corresponding sites of pNL4-3 as described previously [32]. The sequence analysis of adapted viral clones was done for the region from *Sbf*I to *Bsa*BI sites.

### 2.4. Replication Assays

Virus stocks were prepared and quantified as described above. Equal amounts of virus (10^4^ RT units) were inoculated into H9 cells (10^5^ cells). Culture supernatants of the infected H9 cells were collected every three days to monitor virus replication. Single-cycle infectivity assays were performed as previously described [29]. Equal amounts of viruses (10^4^ RT units) were inoculated into TZM-bl cells and on day 2 post-inoculation, the cells were lysed and subjected to luciferase assays.

### 2.5. Western Blotting Analysis

HEK293T cells were transfected with proviral clones or pcNLmini-RI-based clones. On day 1 post-transfection, the cells were harvested and lysed for Western blotting analysis. Western blotting analyses using anti-HIV-1 Vif 319 (catalog no. ab66643; Abcam, Tokyo, Japan) and anti-β-actin clone AC-15 (Sigma-Aldrich, Burlington, MA, USA) antibodies were performed as described previously [29,32].

### 2.6. Semiquantitative RT-PCR Analysis of Splicing Products

For analysis of splicing products using the pcNLmini-RI vector, semiquantitative RT-PCR analysis was carried out similarly as described previously [29,32]. Briefly, HEK293T cells were transfected with vectors, and on the next day, the cells were lysed for automatic RNA extraction using a QIAamp Viral RNA Mini kit (Qiagen, Hilden, Germany) and QIAcube system (Qiagen). RNA samples were used for cDNA synthesis using an oligo(dT) primer. Semiquantitative PCR reactions were performed using the cDNA samples as templates and specific primer sets for all transcripts, the full and D1/A1 products, and the D1/A1-D2b/A2, D1/A1-D2/A2, and D1/A2 products [29,32]. PCR amplicons were analyzed by the agarose gel electrophoresis using Metaphor agarose (Lonza Group AG, Basel, Switzerland) followed by visualization using the Amersham Imager 600 instrument (GE Healthcare, Chicago, IL, USA).

## 3. Results

### 3.1. Extremely Low Vif-Type NL-Y226tac Acquired an Adaptive Mutation That Increases Vif Expression and Viral Replication Potential during a Long-Term Infection in High A3G-Expression Cells H9

By long-term cultures of A3G highly expressing H9 cells infected with excessive and low Vif-types of HIV-1 clones, we have successfully identified adaptive mutations within the SA1D2prox that optimize Vif expression level and increase viral replication ability [32]. HIV-1 adaptation experiments are useful tools to evaluate mutations/adaptation required for optimal viral replication. We have previously shown that an HIV-1 clone NL-Y226tac exhibits a drastic decrease in Vif expression level and a significantly attenuated growth phenotype in H9 cells compared to WT (NL4-3), thus being designated ultra-low Vif-type [29]. In order to investigate how the low Vif-type HIV-1 clone can mutate and adapt to highly restrictive environments, the NL-Y226tac virus was prepared by transfection and inoculated into H9 cells with a ten-fold higher amount than WT (Figure 2A Experiment 1). While NL-Y226tac replication was undetectable at the beginning, as determined by RT activity released into culture supernatants, it was observed from around 60 days post-infection and, thereafter, during the prolonged culture maintained by adding fresh uninfected H9 cells. On day 142 post-infection, virus replication reached a peak in this experiment. In order to construct potentially growth-adapted virus clones, the culture supernatants harvested on days 94 and 142 were newly infected into H9 cells, and the infected cells were collected for DNA extraction at the peak of virus replication. Proviral genomes in the cells were amplified by PCR using the DNA samples as templates and cloned into WT NL4-3 using unique *Sbf*I and *Bsa*BI sites in its genome to generate proviral clones (Figure 1 and Figure 2A). Resultant viral clones derived from culture supernatants on days 94 and 142 were named NL-Ad1 and NL-Ad2, respectively (Figure 2A).

**Figure 3:**
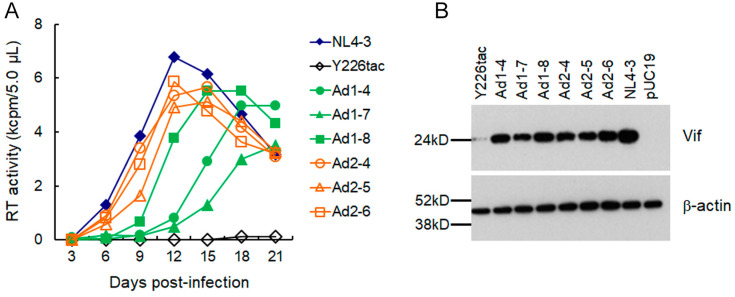
Properties of various clones. Replication properties and Vif expression of control (WT NL4-3 and parental Y226tac) and adapted clones are shown. (**A**) Growth kinetics. Viruses prepared from HEK293T cells transfected with the indicated proviral clones were inoculated into H9 cells. Viral replication was monitored using virion-associated RT activity in the culture supernatants. Representative data from two independent experiments are shown. (**B**) Vif expression levels. HEK293T cells were transfected with the indicated clones, and on day 2 post-transfection, cell lysates for Western blotting analysis using anti-Vif and anti-ß-actin antibodies were prepared. An empty vector pUC19 was used as a negative control.

We then monitored about ten clones for each of NL-Ad1 and NL-Ad2 for their replication abilities. While some of them were replication-incompetent in H9 cells during the observation period, replication-competent clones (NL-Ad1-4, -7, and -8 and NL-Ad2-4, -5, and -6) were obtained and used for further virological analyses. To compare the growth ability, virus samples prepared from these “adapted” clones, WT NL4-3, and NL-Y226tac were inoculated into H9 cells with an equal viral amount. As shown in Figure 3A, while virus replication of a parental NL-Y226tac was negligibly observed during the observation period, all six adapted clones tested grew well in H9 cells but not beyond the WT level. Generally, the NL-Ad2 clones appeared to grow better than the NL-Ad1 clones. We next determined the Vif expression level in transfected HEK293T cells of the proviral clones used for multi-cycle replication assays (Figure 3B). Although somewhat varied among the clones tested, all the six clones consistently expressed a much higher level of Vif relative to parental NL-Y226tac and also a significantly lower level than WT (Figure 3B). These results show that the excessive low Vif-type NL-Y226tac was able to acquire mutation(s) to adapt to highly restrictive environments by increasing the Vif expression, thereby enhancing viral growth potential.

### 3.2. A Mutation (g5061a) within the SD2b Site Is an Adaptive Mutation Responsible for Increasing Vif Expression and Viral Replication Ability

**Figure 4:**
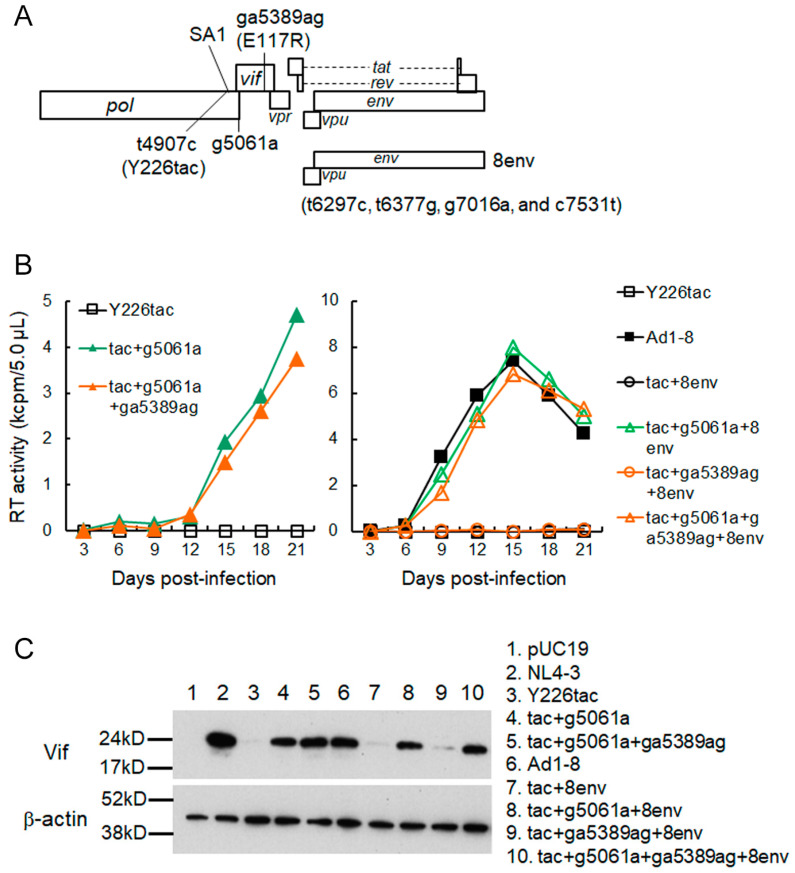
Identification of an adaptive mutation responsible for an increase in Vif expression level. (**A**) Mutations analyzed for an adapted clone (NL-Ad1-8). Region from *vpu* to *env* of NL-Ad1-8 carries four mutations (8env). The SA1 site is indicated. (**B**) Growth kinetics. Various clones constructed were transfected into HEK293T cells to prepare input virus samples for infection. H9 cells were infected with equal amounts (RT) of viruses and virus replication was monitored using RT activity in the culture supernatants. (**C**) Vif expression levels. HEK293T cells were transfected with the indicated clones and the cell lysates were used for Western blotting analysis. Clones analyzed are shown as indicated. Plasmids pUC19 and pNL4-3 were used as negative and positive controls, respectively.

Our adapted NL-Ad1 and NL-Ad2 clones exhibited increased Vif expression levels and growth potentials to various extents relative to a parental NL-Y226tac clone (Figure 3). First, in order to identify adaptive mutation(s) responsible for the increase in Vif expression, adapted clones (NL-Ad1-4, -7, and -8 and NL-Ad2-4, -5, and -6) were sequenced (Table 1 and Table 2). All six clones showed the emergence of mutations all over their whole genomes. Of these mutations, Y226tac (an initial original mutation), g5061a (for Vif-frame, V7/for Pol-IN-frame, D278N), and ga5389ag (for Vif-frame, E117R) were common among the genomes of all adapted clones. Since an increase in Vif expression level of an ultra-low Vif-type clone Y226tac is essential for its readily recognizable replication property in H9 cells, we predicted that some common mutation(s) found could be related to augmenting the Vif expression level. In order to determine which mutation(s) or its combination(s) is responsible for the enhancement of Vif expression and replication ability, we constructed various clones using NL-Ad1-8 with the highest growth ability in the NL-Ad1 group as a positive control for comparison (Figure 3 and Figure 4). We first generated NL-Y226tac clones carrying g5061a or g5061a plus ga5389ag. The mutation g5061a enhanced viral replication ability as well as Vif expression level compared to NL-Y226tac, whereas ga5389ag did not result in a further significant increase in both viral growth and Vif expression (Figure 4B left,C). We then examined a series of NL-Y226tac clones that carry the four mutations within the *vpu-env* region (tac+8env) or combined mutations with g5061a/ga5389ag (tac+g5061a+8env, tac+ga5389ag+8env, and tac+g5061a+ga5389ag+8env) (Figure 4). No appreciable increases in virus replication ability and Vif expression level were observed for the clones without g5061a mutation (Figure 4B right,C). In sharp contrast, the clones carrying g5061a grew better and expressed more Vif relative to parental NL-Y226tac, displaying the virus replication and Vif expression at a level comparable to an adapted NL-Ad1-8 clone (Figure 4B right,C). The results here indicate that g5061a is the adaptive mutation that contributes to the enhancement of Vif expression level and viral growth potential of an ultra-low Vif-type NL-Y226tac clone.

**Figure 5:**
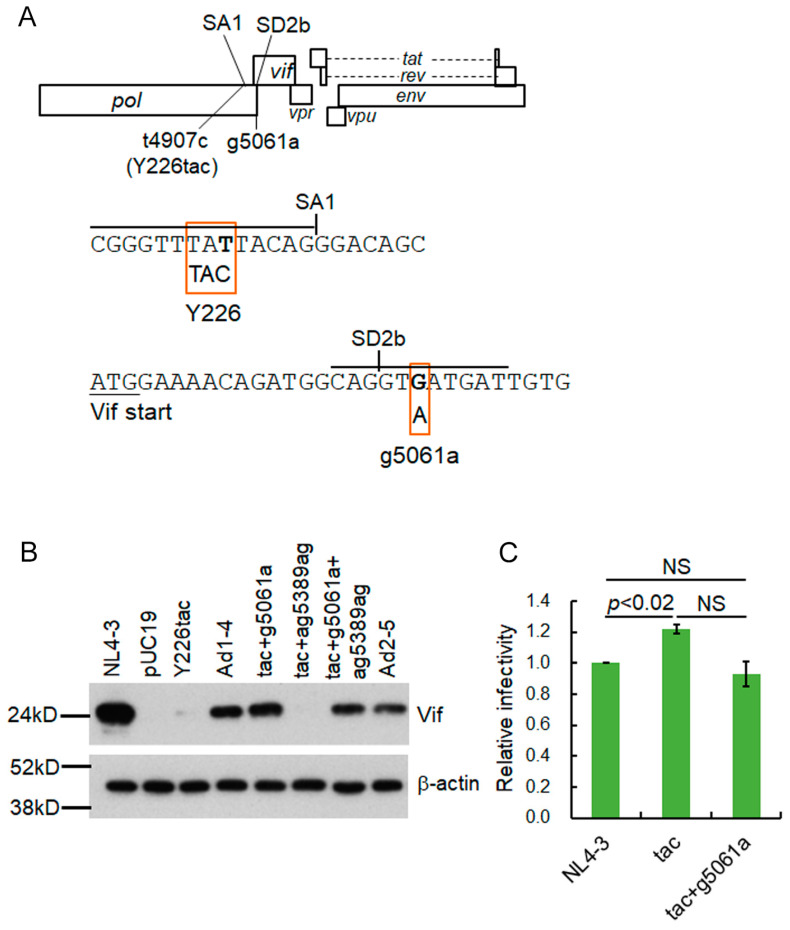
Effect of an adaptive mutation g5061a within SD2b on Vif expression and viral early infectivity. (**A**) Schematic representation and sequences of parental mutation Y226tac (SA1 site) and adaptive mutation g5061a (SD2b site). Mutations within the sequence are highlighted by orange squares. The Vif start codon is indicated. (**B**) Vif expression levels. Cell lysates prepared from HEK293T cells transfected with the indicated proviral clones were analyzed for Vif and cellular ß-actin expression by immunoblotting. (**C**) Viral infectivity. Viruses prepared from HEK293T cells transfected with the indicated clones were inoculated into TZM-bl cells, and on day 2 post-inoculation, cells were lysed for luciferase assays. Infectivity is presented as luciferase activity relative to that exhibited by NL4-3. Mean values ± standard errors (SE) from three independent experiments are shown. Significance relative to NL4-3 was determined by Welch’s *t*-test. NS, not significant.

The adaptive g5061a mutation is located within the rarely used SD2b site in the viral genome (Figure 5A) [24,27]. In order to further confirm that only g5061a mutation, but not ga5389ag mutation, is responsible for the increase in Vif expression level of NL-Y226tac, we generated NL-Y226tac harboring either g5061a or ga5389ag (tac+g5061a and tac+ga5389ag, respectively). As shown in Figure 5B, Vif expression level was not affected by the introduction of ga5389ag mutation, whereas g5061a alone did increase the Vif expression level in NL-Y226tac. Adapted clones NL-Ad1-4 and NL-Ad2-5 exhibited similar expression levels of Vif to that of tac+g5061a clone. Since the g5061a mutation results in an amino acid mutation in the Pol-IN region (Pol-IN D278N) (Table 1 and Table 2), we assessed the effect of the mutation on virus infectivity (the early replication phase) using a luciferase reporter cell line TZM-bl (Figure 5C). To this end, the experiment was performed under APOBEC3 protein-free condition using TZM-bl cells and virus samples prepared from HEK293T cells transfected with the proviral clones. While NL-Y226tac clone displayed slightly higher infectivity than that of WT NL4-3, virus infectivity of tac+g5061a clone was similar to those of WT NL4-3 and parental NL-Y226tac, suggesting that the amino acid substitution Pol-IN D278N by g5061a mutation does not affect significantly the early phase of replication (Figure 5C). Taken together, these results show that the increase in Vif expression level by acquiring g5061a mutation within the SD2b site leads to the enhancement of growth potential of parental NL-Y226tac.

### 3.3. Another Adaptation Experiment Demonstrated the Importance of a Mutation within SA1 or SD2b for Enhancement of Vif Expression Level

**Figure 6:**
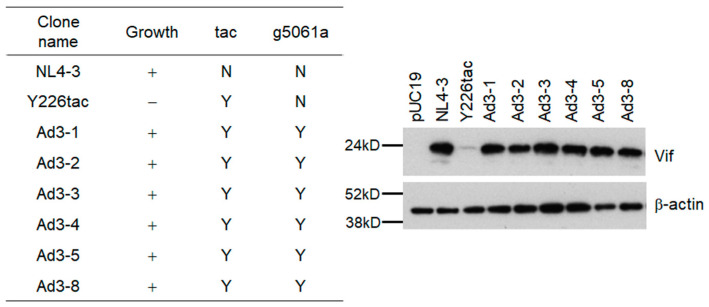
Growth ability and Vif expression level of adapted clones (NL-Ad3). (**Left panel**) For various clones, virus growth in H9 cells during the 21 days post-infection period as monitored using RT assays is indicated as positive (+) or non-detectable (−). In these clones, the mutations tac and g5061a are detected (Y) or not detected (N). (**Right panel**) Vif expression levels of the clones were analyzed as described in Figure 5.

Adaptive mutations critical for viral growth can emerge at a certain frequency under some virus-restrictive conditions. If the g5061a mutation is important for HIV-1 adaptation to alter the *vif* mRNA production, it might appear again in the viral genome in another adaptation experiment. In a newly performed adaptation experiment (Figure 2B, Experiment 2), ten-fold more NL-Y226tac virus was also used for infection to increase the possibility to identify new adaptive mutations. As we expected, virus replication in cells infected with a higher virus dose (Y226tac(H)) was detected earlier than that in those infected with the same dose in the previous experiment (Y226tac) (around 24 days vs. 40 days post-infection). In order to obtain potentially adapted viruses, culture supernatants collected on day 118 post-infection, when both infection groups reached the peak of virus replication, were inoculated into fresh H9 cells (Figure 2B). Adapted viral clones derived from the NL-Y226tac-infection and NL-Y226tac(H)-infection in Figure 2B were constructed as described above, and were named NL-Ad3 and NL-Ad4 clones, respectively. We first examined viral replication abilities of NL-Ad3 clones in H9 cells, and found that nine of ten clones tested were replication-competent though varied in their replication abilities. Of these replication-competent clones, six clones (NL-Ad3-1, -2, -3, -4, -5, and -8) were analyzed for their Vif expression and were demonstrated to exhibit higher levels than the parental NL-Y226tac clone (Figure 6). We selected three clones (NL-Ad3-2, -4, and -8) for sequence analyses (Table 3). While sequences of NL-Ad3-2, and -4 clones were identical, all the three clones harbored the adaptive g5061a mutation we identified above (Table 1 and Table 2, Figure 4 and Figure 5) in spite of several other mutations in their genomes. We then examined and confirmed the presence of the g5061a mutation in all the genomes of other clones (NL-Ad3-1, -3, and -5) (Figure 6). In total, in an independent adaptation experiment, the g5061a mutation emerged frequently and reproducibly, suggesting the importance of this adaptive mutation for increase in the Vif expression level of the parental NL-Y226tac clone.

**Figure 7:**
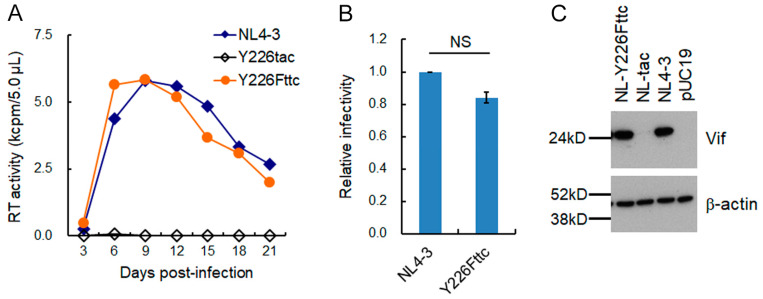
Effect of an adaptive mutation Y226Fttc on viral replication ability, early infectivity, and Vif expression level. (**A**) Growth kinetics. Multi-cycle replication assays using H9 cells were performed as described in Figure 4. (**B**) Infectivity. Viral early infectivity was determined as described in Figure 5. (**C**) Vif expression levels. Vif expression levels of the indicated clones were determined as described in Figure 5.

Newly generated NL-Ad4 clones, which were derived from a long-term culture of cells infected with higher amounts of the virus, were monitored for replication ability in H9 cells. Only two of the six clones tested were replication-competent, but their growth kinetics were similar to that of WT NL4-3. The two clones (NL-Ad4-2 and -4) were sequenced and found to have a common mutation Pol-IN Y226Fttc, which is a single-nucleotide alteration relative to parental Y226tac (Table 4). Thus, a proviral clone carrying only the Pol-IN Y226Fttc (NL-Y226Fttc) mutation was newly generated and examined for its replication ability in H9 cells. As shown in Figure 7A, the NL-Y226Fttc clone showed similar growth potential to WT NL4-3, completely recovering from severely attenuated replication ability of the parental NL-Y226tac clone. Since Y226Fttc amino acid mutation is located in the Pol-IN region of the HIV-1 genome, we asked whether the mutation affects viral infectivity at the early replication phase using TZM-bl cells (Figure 7B). No significant difference in viral infectivity between WT NL4-3 and NL-Y226Fttc was observed. Importantly, Vif expression level of NL-Y226Fttc dramatically increased up to a similar level to that of WT NL4-3 (Figure 7C), indicating that a single-nucleotide alteration Y226Fttc from parental Y226tac is an adaptive mutation. Taken all together, our results demonstrated that the ultra-low Vif-type NL-Y226tac clone acquires an enhanced replication ability by increasing its Vif expression level through adaptive mutations either g5061a in SD2b or Y226Fttc in SA1. Furthermore, reproducible and relatively frequent identification of the g5061a mutation in adapted proviral clones suggests the importance of the SD2b splicing site in modulating the *vif* mRNA production/Vif expression level.

### 3.4. Adaptive Mutations g5061a and Y226Fttc Enhance the vif mRNA Production by Increasing the Splicing Site Usage of SD2b and SA1, Respectively

**Figure 8:**
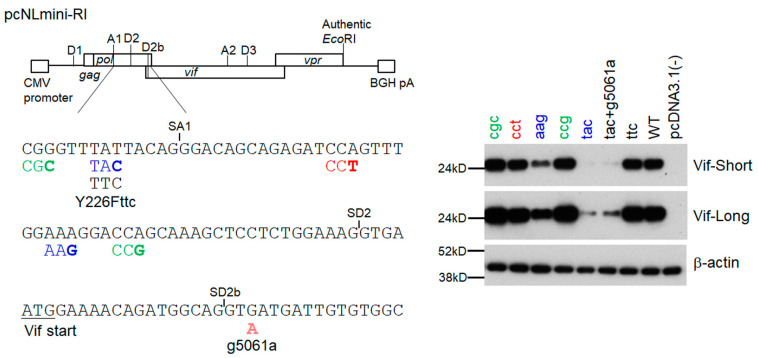
Effect of various single-nucleotide mutations within the region around SA1 and SD2b on Vif expression levels of minigenome (pcNLmini-RI) constructs. (**Left panel**) Genome organization of the pcNLmini-RI vector [32] is presented along with various splicing sites, *Eco*RI site within Vpr-coding region, 5′ CMV promoter, and 3′ BGH poly A (pA). Nucleotide sequence of the region is shown. Mutations in low Vif-type, high Vif-type, and excessive Vif-type are indicated by blue, red, and green letters, respectively. Splicing sites and adaptive mutations (g5061a and Y226Fttc) are shown. (**Right panel**) Vif expression levels of minigenome constructs that carry the indicated mutations were analyzed as described in Figure 5. WT, wild type; Short, short exposure; Long, long exposure.

To analyze changes in the *vif* mRNA production pattern by adaptive mutations within the SD2b and SA1 sites, we used a minigenome previously constructed [32]. Our minigenome (pcNLmini-RI) mainly consists of the authentic NL4-3 nucleotide sequence (pcNLmini-RI in Figure 8), and thus its Vif expression level, as determined by single-nucleotide mutations within the SA1D2prox region, fluctuates in an essentially same manner to that for a proviral clone NL4-3 [32]. We constructed some minigenome constructs containing the initial mutation Y226tac (tac) and/or adaptive mutations (g5061a and Y226Fttc) to monitor their Vif expression (Figure 8). As controls, excessive (cgc and ccg), high (cct), low (aag), and ultra-low (tac) Vif-type [29,30] minigenome clones were also used. These minigenome constructs were transfected into HEK293T cells and examined for their Vif expression. As shown in Figure 8, all minigenome constructs including control and adaptive mutant clones showed the Vif expression pattern as expected. While Vif expression level in the tac clone was dramatically reduced compared to WT, the g5061a introduction into this clone (tac+g5061a) led to an increase in Vif expression level. As for the clone substituting parental tac to adaptive ttc, its Vif expression level was enhanced to that of WT as shown for the proviral clones (Figure 7 and Figure 8).

**Figure 9:**
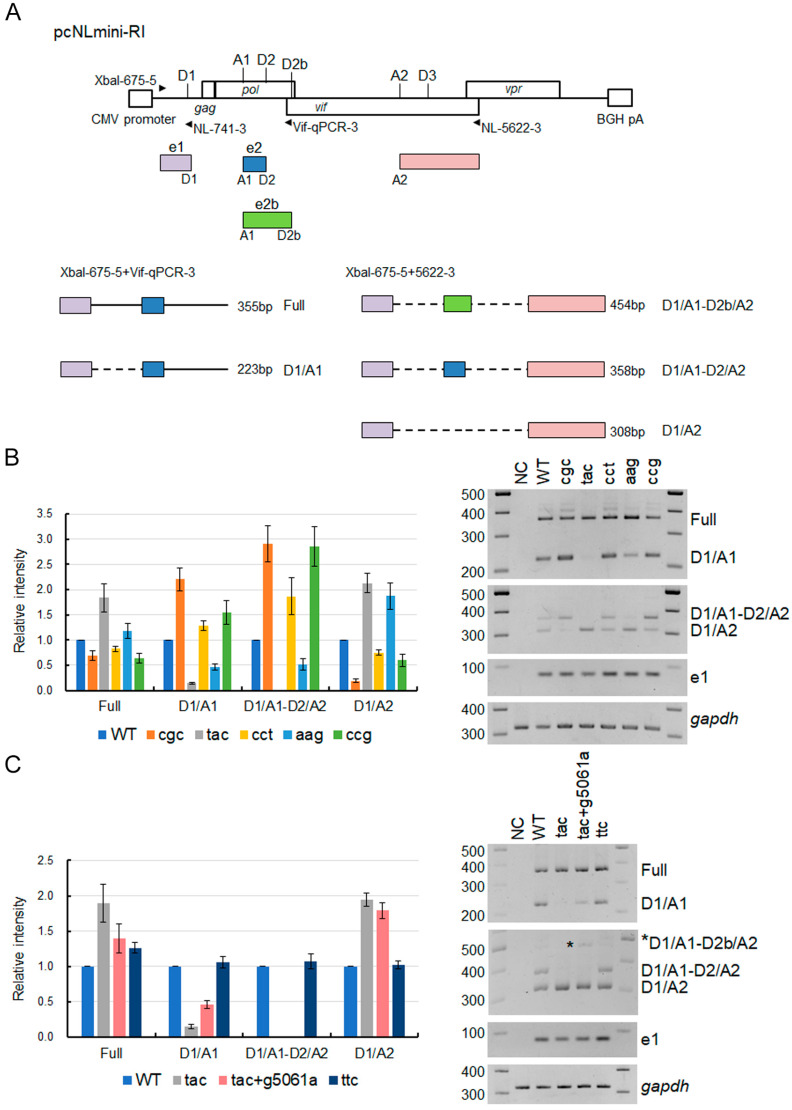
Effect of various mutations within the region around SA1 and SD2b on viral mRNA production. (**A**) Genome organization and splicing products of the minigenome (pcNLmini-RI). Splicing sites and exons are indicated. Primers used for semiquantitative RT-PCR analysis are shown by arrow heads and their names [32]. PCR products (Full, D1/A1, D1/A1-D2b/A2, D1/A1-D2/A2, and D1/A2) amplified using specific primer sets are presented along with the organization and length. (**B**,**C**) Effect of single-nucleotide mutations within the SA1D2prox region (**B**) and adaptive mutations (**C**) on the splicing pattern of the minigenome constructs. HEK293T cells were transfected with pcNLmini-RI vectors carrying the indicated mutations and on the next day, cell lysates were prepared for semiquantitative RT-PCR analysis using primer sets shown in (**A**). Signal intensities of PCR products were quantitated from three independent experiments. The intensities of the indicated mRNAs in each sample were normalized to those of all viral mRNAs (e1) and cellular *gapdh* mRNA. The normalized mRNA intensities in each sample relative to those of WT are presented.

Since adaptive mutations (g5061a and Y226Fttc) were located within the splicing sites SD2b and SA1, respectively, we calculated the splicing site usage by the Hbond score for the SD2b site and by the MaxEnt score for the SA1 site (Table 5). The adaptive mutation g5061a increased the Hbond score relative to that of WT (15.8 vs. 12.4). The MaxEnt score at the SA1 site was decreased in Y226tac compared to WT, whereas Y226Fttc showed a higher score than that of WT in all three different calculation models (Table 5). These results suggested that adaptive mutations g5061a and Y226Fttc increase the splicing site usage at the SD2b and SA1, respectively. In order to experimentally confirm the changes in splicing site usage, mRNA species produced through the utilization of various splicing sites were analyzed by semiquantitative PCR using the minigenome constructs (Figure 9). HEK293T cells were transfected with minigenome constructs and processed as described previously for semiquantitative RT-PCR analysis [29,32] using specific primer sets to detect various splicing products (Full, D1/A1, D1/A1-D2b/A2, D1/A1-D2/A2, and D1/A2) depending on their lengths (Figure 9A). Of these products, the D1/A1 product corresponds to *vif* mRNA. As for control mutants (Figure 9B), excessive (cgc and ccg) and high (cct) Vif-type constructs exhibited higher levels of D1/A1 and D1/A1-D2/A2 products than WT because of high SA1 site usage, whereas the D1/A2 products were reduced in these constructs. The opposite effects were observed for low (aag) and ultra-low (tac) Vif-type constructs. The results obtained here (Figure 9B) were consistent with previous reports by us and others that show the inverse correlation between SA1 and SA2 usages [29,30,39]. As shown in Figure 9C, while the tac mutation significantly decreased the D1/A1 product, our newly identified adaptive mutation Y226Fttc recovered D1/A1 mRNA production. The splicing pattern of the ttc construct was very similar to that of WT. Another adaptive mutation g5061a increased the D1/A1 product as well as the D1/A1-D2b/A2 product relative to the parental tac construct, indicating simultaneous elevation in SD2b and SA1 site usage by the mutation (Figure 9C). In total, these results showed that the decreased *vif* mRNA production by the tac mutant can be reduced or canceled through increased usage of SD2b and/or SA1 sites by adaptive mutations.

## 4. Discussion

In this study, through long-term infection experiments in highly A3G-expressing lymphocytic H9 cells, we investigated whether an ultra-low Vif-type HIV-1 clone with a minimum growth ability (Y226tac) can adapt itself to replicate well under the restrictive condition by increasing the Vif expression. We were also interested to know how an adaptive mutation(s) influences the expression pattern of the *vif* mRNA. Moreover, we aimed to address identifying viral genomic region(s) that can contribute to the modulation of the Vif expression level by thorough analysis of adaptive mutations emerged.

During adaptation experiments (long-term cultures of Y226tac-infected H9 cells), viruses that potentially have higher replication ability than the parental clone emerged. Of adapted virus clones generated, six replication-competent clones grew much better than the parental Y226tac clone with a severely attenuated phenotype in H9 cells. These six clones with an augmented replication potential displayed increased Vif expression levels with no exception. Sequence analysis showed that while mutations appeared in their overall genomes, all six adapted clones carried parental tac in SA1 and g5061a mutation within the SD2b site. Indeed, the g5061a mutation was an adaptive mutation responsible for the increased Vif expression level of the six clones. In an independently performed adaptation experiment, the g5061a mutation repeatedly and reproducibly emerged in the genomes of adapted clones, indicating the importance of this mutation in the adaptation process of an ultra-low Vif-type Y226tac clone. Furthermore, we identified another adaptive mutation Y226Fttc within the SA1 site, which is a single-nucleotide substitution of parental Y226tac, in the adaptation experiment using a higher input dose of the Y226tac virus. The Y226Fttc mutation recovered Vif expression level as well as replication ability to those of WT NL4-3. The splicing site usage of the SD2b and SA1 sites was predicted to be increased by adaptive mutations g5061a and Y226Fttc, respectively. The splicing pattern analyzed using minigenome constructs was changed by adaptive mutations in an inferred way. The g5061a mutation activated splicing at the SD2b site as judged by the increase in D1/A1-SD2b/A2 products. Probably due to the enhancement of the SA1 site usage to the WT level, Y226Fttc showed a similar splicing pattern with WT. Taken together, these results showed that even an ultra-low Vif-type virus can alter splicing efficiency by a single-nucleotide adaptive mutation acquired under the strictly restricted condition, thereby improving its replication ability via the increase in Vif expression level.

Regarding adapted clones obtained from the long-term culture (Experiment 1), we noticed that NL-Ad2 clones grew better than NL-Ad1 clones. It is conceivable that mutations that emerged in the genomes of NL-Ad2 clones may contribute to the enhancement of viral growth. In fact, we have previously shown that the E427K mutation in Env-gp120, which is found in NL-Ad2-6 but not in any NL-Ad1 clones, increases viral replication ability [40]. Efforts are currently ongoing to determine adaptive mutations that enhance viral growth potential in NL-Ad2 clones in our laboratory. The emergence of adapted viruses in our experiments demonstrates that even HIV-1 expressing a quite low amount of Vif can survive under exceedingly restrictive conditions imposed by high levels of APOBEC3 proteins. Interestingly, G-to-A mutations that are a hallmark for the mutagenic activity of APOBEC3G were frequently found in the adapted clones tested. This implies that without complete suppression of HIV-1 replication by APOBEC3 proteins, HIV-1 can utilize their mutagenic activities to acquire adaptive mutations. In other words, as reported previously but is still controversial [41,42,43,44,45,46,47], these results suggest that mutations by APOBEC3 proteins may drive HIV-1 adaptation/evolution, especially in the cell culture systems.

In our long-term infection experiments, we found two adaptation pathways of an ultra-low Vif-type NL-Y226tac clone, which augment viral replication ability by increasing Vif expression level through alteration in the splicing efficiency at the SD2b and SA1 sites. In one pathway, an adaptive Pol-IN Y226Fttc mutation was located within the SA1 site, which is a single-nucleotide substitution of parental Y226tac, but not a revertant to WT NL4-3 Y226tat. This amino acid mutation Pol-IN Y226F did not affect the early phase of replication which strongly links to the Pol-IN function. Rather, the single-nucleotide change in Y226Fttc resulted in an enhanced Vif expression level to the WT level by elevating the splicing at the SA1 site, and thereby augmented viral growth potential. In another pathway, the SD2b site where an adaptive g5061a mutation emerged is known to be a rarely used site [12,24,48]. While the intrinsic strength of the SD2b site is higher than that of the SD2 site as calculated by the Hbond score, the SD2 site is more frequently utilized for splicing than the SD2b site in the context of the viral genome [24]. Although the splicing at the SD1 and SA1 sites is essential to produce the *vif* mRNA, at the same time, the usage of downstream SD2 and SD2b sites inhibits the *vif* mRNA production. On the other hand, the recognition of the SD2 and SD2b by U1snRNP activates the splicing at the SA1 site by inducing the binding of U2snRNP [24,27]. The *vif* mRNA level optimal for viral replication is determined by various factors involved in the complicated regulation such as the splicing efficiency of SD and SA sites, SREs, and splicing regulatory factors [10,11,12,13,14,15,16,24,25,26,27]. In our ultra-low Vif-type Y226tac, the splicing acceptor strength at the SA1 site was predicted to be reduced by the tac mutation compared to WT. While several SREs that inhibit the splicing are located around the SD2b site, it is conceivable that the g5061a mutation activated the usually rarely used SD2b site and induced subsequent splicing activation of the SA1 site. Given the frequent and reproducible emergence of the g5061a mutation in our experiments, the increase in the splicing site usage at the SD2b would be a key adaptation process of rescuing the decreased *vif* mRNA production through the splicing activation of the SA1 site.

Various *cis*-acting elements including packaging, splicing, and SREs are present on genomes of RNA viruses [14,49,50,51,52], and mutations in these and also in unknown elements can affect virus replication. Recent research progress on the biological relevance and effect of dinucleotide frequency, codon usage, RNA modification, and RNA structure [9,52,53,54,55,56,57,58] suggests that there can be more unknown adaptation processes of viruses in a nucleotide sequence-dependent manner. In order to more deeply understand the adaptation of RNA viruses, further studies are required to uncover the role and significance of the genome sequence, modification, and structure in virus replication and adaptation.

## Figures and Tables

**Table 1:** Mutations found in adapted clones (NL-Ad1 clones).

NL-Ad1-4			NL-Ad1-7			NL-Ad1-8		
Nt Change	Region	NS/S Change in the Region	Nt Change	Region	NS/S Change in the Region	Nt Change	Region	NS/S Change in the Region
						t3707c	Pol-RT	T386acc
t4907c	Pol-IN	Y226tac	t4907c	Pol-IN	Y226tac	t4907c	Pol-IN	Y226tac
g5061a	Vif	V7	g5061a	Vif	V7	g5061a	Vif	V7
g5061a	Pol-IN	D278N	g5061a	Pol-IN	D278N	g5061a	Pol-IN	D278N
			g5224a	Vif	A62T			
ga5389ag	Vif	E117R	ga5389ag	Vif	E117R	ga5389ag	Vif	E117R
a5627g	Vpr	L23ttg	a5627g	Vpr	L23ttg			
						t6297c	Vpu	D79
						t6297c	Env(SP)	M26T
g6308a	Env(SP)	A30T						
						t6377g	Env(C1)	F53V
g6443a	Env(C1)	V75I						
g6654a	Env(V1)	G145E						
						g7016a	Env(C2)	E266K
g7505a	Env(C4)	G429R						
						c7531t	Env(C4)	I437att

Nt, nucleotide; NS, nonsynonymous; S, synonymous. Words in parentheses show the Env domains. SP, signal peptide.

**Table 2:** Mutations found in adapted clones (NL-Ad2 clones).

NL-Ad2-4			NL-Ad2-5			NL-Ad2-6		
Nt Change	Region	NS/S Change in the Region	Nt Change	Region	NS/S Change in the Region	Nt Change	Region	NS/S Change in the Region
c3058t	Pol-RT	P170L	c3058t	Pol-RT	P170L	c3058t	Pol-RT	P170L
t4907c	Pol-IN	Y226tac	t4907c	Pol-IN	Y226tac	t4907c	Pol-IN	Y226tac
g5061a	Vif	V7	g5061a	Vif	V7	g5061a	Vif	V7
g5061a	Pol-IN	D278N	g5061a	Pol-IN	D278N	g5061a	Pol-IN	D278N
ga5389ag	Vif	E117R	ga5389ag	Vif	E117R	ga5389ag	Vif	E117R
						g6273a	Vpu	G71gga
						g6273a	Env(SP)	G18D
g6443a	Env(C1)	V75I	g6443a	Env(C1)	V75I	g6443a	Env(C1)	V75I
g7160a	Env(V3)	A314T						
						t7282a	Env(C3)	N354K
			t7408a	Env(V4)	S396R			
						g7499a	Env(C4)	E427K

Nt, nucleotide; NS, nonsynonymous; S, synonymous. Words in parentheses show the Env domains. SP, signal peptide.

**Table 3:** Mutations found in adapted clones (NL-Ad3 clones).

NL-Ad3-2			NL-Ad3-4			NL-Ad3-8		
Nt Change	Region	NS/S Change in the Region	Nt Change	Region	NS/S Change in the Region	Nt Change	Region	NS/S Change in the Region
g3839a		E430gaa	g3839a		E430gaa	g3839a	Pol-RT	E430gaa
						g4080a	Pol-RT	D511N
t4907c	Pol-IN	Y226tac	t4907c	Pol-IN	Y226tac	t4907c	Pol-IN	Y226tac
g5061a	Vif	V7	g5061a	Vif	V7	g5061a	Vif	V7
g5061a	Pol-IN	D278N	g5061a	Pol-IN	D278N	g5061a	Pol-IN	D278N
						a5447t		Q136L
g5484a	Vif	L148tta	g5484a	Vif	L148tta			
						t6069c	Vpu	P3ccc
						g6150a	Vpu	R30aga
						g6225a	Vpu	E55gaa
						g6225a	Env(SP)	R2K
						g6229a	Vpu	E57K
						g6229a	Env(SP)	V3gta
						a6513t	Env(C1)	N98I
g6845a	Env(C2)	E209K	g6845a	Env(C2)	E209K			
						g7499a	Env(C4)	E427K
a7507g	Env(C4)	G429ggg	a7507g	Env(C4)	G429ggg			

Nt, nucleotide; NS, nonsynonymous; S, synonymous. Words in parentheses show the Env domains. SP, signal peptide.

**Table 4:** Mutations found in adapted clones (NL-Ad4 clones).

NL-Ad4-2			NL-Ad4-4		
Nt Change	Region	NS/S Change in the Region	Nt Change	Region	NS/S Change in the Region
a4906t	Pol-IN	Y226Fttc	a4906t	Pol-IN	Y226Fttc
g5138t	Vif	R33M			
			c5512a	Vif	Q158K
c5622t	Vpr	L22F			
g5672a	Vpr	W38stop			
			g5788a	Vpr	R77Q
t6584c	Env(C1)	L122cta			
c6830t	Env(C2)	P204S	c6830g(C2)	Env	P204S
g6907a	Env(C2)	K229aag			

Nt, nucleotide; NS, nonsynonymous; S, synonymous. Words in parentheses show the Env domains.

**Table 5:** Effect of mutations on the splicing site usage.

					MaxEnt Score		
Mutation	Sequence around the SD2b Site	Hbond Score	Mutation	Sequence around the SA1 Site	ME	MM	WMM
WT	CAGGTGATGAT	12.4	WT	AATTTTCGGGTTTATTACAGGGA	6.41	7.07	7.00
g5061a	CAGGT**A**ATGAT	15.8	Y226tac	AATTTTCGGGTTTA**C**TACAGGGA	5.92	5.72	6.90
			Y226Fttc	AATTTTCGGGTTT**T**CTACAGGGA	8.07	7.48	9.19

Hbond score was calculated on https://rna.hhu.de/HBond/ (accessed on 7 June 2023); MaxEnt score was calculated on http://hollywood.mit.edu/burgelab/maxent/Xmaxentscan_scoreseq_acc.html (accessed on 7 June 2023). ME, maximum entropy model; MM, first-order Markov model; WMM, weight matrix model.

## Data Availability

Research data are available from M.N. upon reasonable request.
